# Early‐Onset Colorectal Cancer With Liver‐Only Metastases: A Retrospective Cohort Study Integrating Prospectively Collected Real‐World Clinical and Molecular Data From an Australian National Database (2009–2024) to Guide Treatment Planning

**DOI:** 10.5694/mja2.70266

**Published:** 2026-08-18

**Authors:** Savio G. Barreto, Christos S. Karapetis, Shahid Ullah, Matthew Burge, Susan Caird, Angus Campbell, Azim Jalali, Ross Jennens, Muhammad A. Khattak, Belinda Lee, Stephanie H. Lim, Shehara Mendis, Louise Nott, Timothy J. Price, Jeremy D. Shapiro, Jeanne Tie, Javier Torres, Colin Williams, Rachel Wong, Vanessa Wong, Peter Gibbs

**Affiliations:** ^1^ Flinders Medical Centre Adelaide South Australia Australia; ^2^ Flinders Health and Medical Research Institute Flinders University Adelaide South Australia Australia; ^3^ Flinders Centre for Innovation in Cancer Flinders Medical Centre Adelaide South Australia Australia; ^4^ Royal Brisbane and Women's Hospital Brisbane Queensland Australia; ^5^ Gold Coast University Hospital Gold Coast Queensland Australia; ^6^ Walter and Eliza Hall Institute of Medical Research Melbourne Victoria Australia; ^7^ Northern Health Melbourne Victoria Australia; ^8^ Latrobe Regional Hospital Traralgon Victoria Australia; ^9^ Epworth Healthcare Melbourne Victoria Australia; ^10^ Fiona Stanley Hospital Perth Western Australia Australia; ^11^ Edith Cowan University Perth Western Australia Australia; ^12^ Peter MacCallum Cancer Centre Melbourne Victoria Australia; ^13^ Macarthur Cancer Therapy Centre Campbelltown Hospital Sydney New South Wales Australia; ^14^ Royal Hobart Hospital Hobart Tasmania Australia; ^15^ Queen Elizabeth Hospital Adelaide South Australia Australia; ^16^ Basil Hetzel Institute Adelaide South Australia Australia; ^17^ Adelaide University Adelaide South Australia Australia; ^18^ Cabrini Health Melbourne Victoria Australia; ^19^ University of Melbourne Melbourne Victoria Australia; ^20^ Goulburn Valley Health Shepparton Victoria Australia; ^21^ Eastern Health Melbourne Victoria Australia; ^22^ Epworth HealthCare Melbourne Victoria Australia; ^23^ Monash University Melbourne Victoria Australia; ^24^ Western Health Melbourne Victoria Australia

**Keywords:** cancer, carcinoma, chemotherapy, colorectal neoplasms, digestive system neoplasms, liver neoplasms, surgical oncology

## Abstract

**Objective:**

To leverage the Treatment of Recurrent and Advanced Colorectal Cancer (TRACC) registry (an Australian cancer database) to explore the ideal timing and sequence of therapies and the factors influencing these decisions in colorectal cancer (CRC) patients with liver‐only metastases to inform contemporary decision‐making and future trials.

**Study Type:**

Retrospective registry‐based cohort study using the TRACC registry.

**Setting and Participants:**

Consecutive patients with liver‐only metastatic CRC enrolled in the TRACC registry.

**Main Outcome Measures:**

To explore cancer biology, intended treatment at presentation, actual treatment received and the resultant outcomes for early‐onset CRC (EOCRC) (≤ 50 years) and late‐onset CRC (LOCRC) (> 50 years) patients with liver‐only metastases from a real‐world perspective.

**Results:**

Between 14 January 2009 and 2 September 2024, 1691 patients with liver‐only metastatic CRC were enrolled in TRACC. These included 276 EOCRC patients (16.3%) and 1415 LOCRC patients (83.7%). In the EOCRC subset, there were more females (48.2% vs. 34.5%, *p* < 0.001), less comorbidity (Charlson comorbidity index score 0, 90% vs. 59%, *p* < 0.001), more left‐sided primaries (76.1% vs. 65.7%, *p* < 0.001), more synchronous disease (53.3% vs. 42.1%, *p* < 0.001) and *BRAF* V600E mutations (13.9% vs. 8.1%; *p* = 0.010). Overall, EOCRC patients had a longer median survival compared with LOCRC patients (3.20 vs. 2.38 years, *p* < 0.001). For the 662 patients (39.1%) undergoing liver resection, median survival was 5.99 years in EOCRC patients and 5.88 years in LOCRC patients. For all patients and for those undergoing resection, respectively, B‐Raf proto‐oncogene, serine/threonine kinase (*BRAF*) (hazard ratio, 1.97 [*p* < 0.001] and hazard ratio, 2.25 [*p* < 0.001]) and Kirsten rat sarcoma viral oncogene homologue (*KRAS*) mutations were associated with worse outcomes (hazard ratio, 1.29 [*p* < 0.001] and hazard ratio, 1.34 [*p* = 0.003]).

**Conclusion:**

Differences in sex distribution, *BRAF* mutation rates, primary tumour site and overall survival suggest biological differences between EOCRC and LOCRC. Liver resection was associated with improved survival in LOCRC, with the benefits of all therapies varying depending on age, primary tumour site and whether patients presented with synchronous or metachronous liver‐only metastases.

## Plain Language Summary

**The known:**Australia has the highest incidence of early‐onset colorectal cancer (CRC) globally.

**The new:**This study, using real‐world data from an Australian cancer database, demonstrates differences in cancer biology, tumour location and treatment sequence and differences in overall survival between early‐onset CRC and late‐onset CRC patients with liver‐only metastases.

**The implications:**These findings warrant consideration of a selective aggressive approach to managing CRC patients with liver‐only metastases based on disease burden, presence of comorbidities and an understanding of the patient's unique tumour biology.

## 1 Introduction

Globally, there has been an increase in early‐onset cancers affecting the gastrointestinal tract, most pronounced in the colon and rectum [1, 2, 3]. Australia has the highest incidence of early‐onset colorectal cancer (EOCRC) in the world [4]. Our forecasting models have revealed that this rising trend in colorectal cancer (CRC) among individuals aged 15–49 years is going to persist in high socio‐demographic index regions with sex‐specific variations in incidence, mortality and disability‐adjusted life‐year rates [5]. On the flipside, there has been a decrease in CRC rates in older patients [6].

EOCRC is poorly understood. It is unclear whether the cancers in EOCRC patients are biologically different from those with late‐onset CRC (LOCRC) (> 50 years of age), and, in turn, if the current treatment algorithms are as effective for the younger cohort as they are for older patients. These questions have arisen from previous studies in early‐stage disease. Fontana et al. [7] used individual patient data from six trials in the International Duration Evaluation of Adjuvant Chemotherapy (IDEA) database to compare clinical characteristics, treatment adherence, adverse events and outcomes in EOCRC versus LOCRC patients. While patients in the IDEA database with EOCRC had a better performance status, had similar tumour (T) stage, were more likely to complete their planned treatment duration (83.2% vs. 78.2% [*p* < 0.01]) and received a higher treatment dose intensity, they experienced more frequent 3‐year relapses in Stage III compared with the LOCRC cohort (69% vs. 76%; HR, 1.21 [95% confidence interval (CI), 1.07–1.37]; *p* = 0.003). The former also had a higher cancer‐specific mortality rate (for those with high‐risk Stage III disease). This variation in survival, despite receiving the same treatment, was previously reported in a stage‐matched comparison of EOCRC Stage III rectal cancer patients [8] as well as in a recent systematic review [9].

EOCRC patients are more likely to present with de novo metastatic disease (71.2% [365/513] vs. 57.4% [1824/3179]; *p* < 0.0001) and be considered for more aggressive treatments (i.e., resection of metastases, lines of chemotherapy and triplet chemotherapy as first‐line therapy) [10]. In addition, presence of metastases in the liver, the most common site for disease spread in CRC [10], is a critical determinant of overall survival (OS) in this cohort of patients [11]. The ideal management in terms of timing of therapies in patients with CRC and liver‐only metastases has been a topic of ongoing debate owing to a lack of high‐quality evidence [12, 13]. Existing registry and trial datasets rarely synthesise bioinformatics with practice‐pattern data to guide when to operate versus deliver neoadjuvant therapy in early‐onset, liver‐only metastatic CRC.

We hypothesise that there exist biological differences between EOCRC and LOCRC patients with liver‐only metastases with resultant differences in survival that are dependent on the treatment received, such as surgery, chemotherapy and biologic therapy. Thus, leveraging the Treatment of Recurrent and Advanced Colorectal Cancer (TRACC) registry (an Australian cancer database), our study aimed to explore cancer biology, intended treatment at presentation, actual treatment received and the resultant outcomes between EOCRC patients (≤ 50 years) and LOCRC patients (> 50 years) with liver‐only metastases from a real‐world perspective. This information will inform contemporary decision‐making and future trials.

## 2 Methods

This retrospective cohort study used prospectively collected data from the TRACC registry [14] which includes CRC patients from 30 Australian sites and one overseas site (Hong Kong). The study was conducted in accordance with the Strengthening the Reporting of Observational Studies in Epidemiology (STROBE) guidelines [15] (Table S1). Inclusion criteria for our analysis were patients aged 18 years and older with metastatic or Stage IV (liver‐only) CRC, who were enrolled between 14 January 2009 and 2 September 2024. TRACC records consecutive Stage IV cases of CRC; these include recurrent and de novo metastatic disease. Patients with other solid organ metastases and those with incomplete data were excluded. Patients were divided into two cohorts: EOCRC ≤ 50 years and LOCRC > 50 years based on previously published age cut‐offs for early‐onset gastrointestinal cancers [2].

We analysed prospectively collected data from these patients. The two cohorts were compared for patient characteristics: sex reported as per the Sex and Gender Equity in Research guidelines, referring to a set of biological attributes in humans and animals that are associated with physical and physiological features, including chromosomes, gene expression, hormone function and reproductive/sexual anatomy (categorised as female or male, although there is variation in the biological attributes that constitute sex and how those attributes are expressed); performance status; and comorbidities. They were also compared for cancer biology: clinico‐pathologic features (primary site, synchronous vs. metachronous disease and number metastatic sites) and molecular data (B‐Raf proto‐oncogene, serine/threonine kinase [*BRAF*], rat sarcoma [*RAS*] and mismatch repair [MMR] status). In addition, they were compared according to treatment sequence (lines of chemotherapy and metastatic resection) and median OS.

Molecular testing data (Kirsten rat sarcoma viral oncogene homologue [*KRAS*], neuroblastoma *RAS* viral oncogene homologue [*NRAS*], *BRAF* and MMR status) were not available for all patients, particularly for those diagnosed in earlier years (2009–2015, 786/1691 [46.5%]) when routine genomic profiling was not yet widely implemented. Cetuximab, a monoclonal antibody that blocks epidermal growth factor receptor, initially became widely available in the first‐line setting in Australia in 2015, prompting routine molecular testing. Analyses involving molecular markers were conducted using the subset of patients with available data. MMR was tested with immunohistochemistry.

Comorbidity data were calculated using the modified Charlson comorbidity index [16]. Performance status was defined by the Eastern Cooperative Oncology Group (ECOG) scale [17]. A right‐sided cancer was defined as one arising in the caecum, ascending colon, hepatic flexure or transverse colon. Tumours at and beyond the splenic flexure were considered a left‐sided primary and included rectal cancers. Rectal cancer was defined as a tumour with the lower border within 12 cm of the anal verge. A line of therapy was defined as any treatment received before disease progression. Overall survival was calculated from date of diagnosis of metastatic disease until death and was censored at last visit if no event had occurred.

### 2.1 Treatment Sequence Groups

Owing to the complexity of varying treatments offered, including systemic chemotherapy, biologic agents and surgeries (collectively referred to as active treatments) involving the primary and/or liver metastases, the patients were grouped as follows:

- chemotherapy/biological therapy: patients who only received chemotherapy and/or therapy with biologic agents regardless of the intention;
- chemotherapy/biological therapy → surgery: patients who received upfront chemotherapy and/or therapy with biologic agents followed by surgery (of the liver, and the primary if synchronous metastases);
- surgery only: patients who only received surgery;
- surgery → chemotherapy/biological therapy: patients who received upfront surgery (of the liver metastases, and the primary if synchronous metastases) followed by chemotherapy and/or therapy with biologic agents before developing progressive disease; and
- palliation/best supportive care/no treatment.

### 2.2 Data Analysis

Ethics approval for this project was obtained from Melbourne Health Human Research Ethics Committee (HREC/18/MH/28, Project 202408/5). The manner in which participant consent was handled within TRACC has previously been detailed [14].

Patient characteristics were expressed as median and interquartile range (IQR) for skewed data. The Mann–Whitney *U*‐test was used to explore the significance of differences between patients based on their age (i.e., between EOCRC and LOCRC groups). Proportions were presented as percentages of the respective denominator and were compared between groups using a standard χ^2^ test for association, with continuity correction where appropriate. Missing data were assessed for all variables. Analyses were conducted using a complete‐case approach for each variable, with patients included based on the availability of relevant data.

Cox proportional hazards models were used to examine the association between treatment sequence and OS. Treatment sequence was defined as the primary exposure variable. Univariate models were first conducted to assess the association between each variable and survival outcomes. A multivariable Cox proportion hazards model was then constructed to estimate the association between treatment sequence and OS while adjusting for clinically relevant covariates and potential confounders. The covariates included age at the time of first metastatic diagnosis, sex, Charlson comorbidity index score, primary tumour site, stage at diagnosis, number of metastatic sites, mutation status for *KRAS*, *NRAS* and *BRAF*, and MMR status. The estimates were calculated using the likelihood ratio method and were expressed as HRs, with an HR < 1 indicating survival advantage for the exposure group. Proportional hazard assumption was tested by log–log plot of survival and Schoenfeld residuals. A Harrell's C‐statistic was used to explore the predictive discrimination ability of the model. The survival time was calculated from the date of diagnosis of CRC with liver‐only metastases to the date of death, censored for lost to follow‐up or end of follow‐up until the date of last review. Survival curves were evaluated by standard Kaplan–Meier survival curves and patient cohorts were compared by log‐rank test. The two‐sided test was performed for all analyses, 95% CIs were reported, and the level of significance was set at *α* = 0.05. All statistical analyses were conducted using R version 4.4.3. Kaplan–Meier survival curves were generated using the ggsurvplot function from the survminer package, while the Cox proportional hazards models were conducted using the coxph function from the survival package. As a sensitivity analysis, we fitted inverse probability of treatment weighted (IPTW) Cox proportional hazards models within each age group using baseline covariates to reduce measured treatment selection bias.

## 3 Results

Between 14 January 2009 and 2 September 2024, 1691 patients with liver‐only metastatic CRC were enrolled in TRACC. A total of 621 (36.7%) were female, and the median age was 65 years (IQR, 55–75 years). There were 276 patients with EOCRC (16.3%) and 1415 patients with LOCRC (83.7%) in our analysis.

### 3.1 Patient Demographic and Clinicopathological Characteristics

Detailed patient demographic and clinicopathological characteristics are presented in Table 1. The proportion of females was significantly higher in the EOCRC group compared with the LOCRC group (48.2% [133/276] vs. 34.5% [488/1415]; *p* < 0.001). The EOCRC patients were significantly more likely than LOCRC patients to have a higher percentage of a Charlson comorbidity index score of 0–1 (97.1% [264/272] vs. 80.4% [1127/1401]; *p* < 0.001) and an ECOG score of 0–1 (97.4% [265/272] vs. 85.0% [1191/1401]; *p* < 0.001). The EOCRC patients were also significantly more likely than LOCRC patients to have left colon and rectal cancers (76.1% [210/276] vs. 65.7% [929/1415]; *p* < 0.001) and synchronous metastases at presentation (53.3% [147/276] vs. 42.1% [595/1413]; *p* < 0.001). Availability of molecular testing data varied by marker; *KRAS* testing was available for 1228 patients (72.6%), *NRAS* for 930 (55.0%), *BRAF* for 1027 (60.7%) and MMR status for 1110 persons (65.6%). EOCRC patients exhibited a significantly higher proportion of cancers with *BRAF* mutations compared with LOCRC patients (13.9% [30/216] vs. 8.1% [66/811]; *p* = 0.010). However, no differences were noted with *KRAS* mutations, *NRAS* mutations or proficient MMR status between these two cohorts. EOCRC cancer patients appeared to undergo molecular testing more frequently than LOCRC patients (67.0% [185/276] to 87.0% [240/276] across the four markers compared with 52.7% [745/1415] to 69.8% [988/1415]), reflecting differences in testing uptake across the study population.

**TABLE 1:** Demographic and clinicopathological characteristics of patients included in the study.

Characteristics	Overall (*N* = 1691)	Age group		*p*
		≤ 50 years (*n* = 276; 16.3%)	> 50 years (*n* = 1415; 83.7%)	
Age (years), median (IQR)	65.0 (55.0–75.0)	43.0 (37.0–47.5)	69.0 (61.0–76.0)	< 0.001
Sex				< 0.001
Male	1070 (63.3%)	143 (51.8%)	927 (65.5%)	
Female	621 (36.7%)	133 (48.2%)	488 (34.5%)	
Charlson comorbidity index score				< 0.001
0	1072 (64.1%)	245 (90.1%)	827 (59.0%)	
1	319 (19.1%)	19 (7.0%)	300 (21.4%)	
2+	282 (16.9%)	8 (2.9%)	274 (19.6%)	
Unknown^a^	18	4	14	
ECOG score				< 0.001
0	866 (51.8%)	202 (74.3%)	664 (47.4%)	
1	590 (35.2%)	63 (23.2%)	527 (37.6%)	
2+	217 (13.0%)	7 (2.6%)	210 (15.0%)	
Unknown	18	4	14	
Primary sites				< 0.001
Left colon	687 (40.6%)	128 (46.4%)	559 (39.5%)	
Right colon	512 (30.3%)	66 (23.9%)	446 (31.5%)	
Rectum	452 (26.7%)	82 (29.7%)	370 (26.1%)	
Others	40 (2.4%)	0 (0.0%)	40 (2.8%)	
Stage at diagnosis				0.001
Metachronous	947 (56.1%)	129 (46.7%)	818 (57.9%)	
Synchronous	742 (43.9%)	147 (53.3%)	595 (42.1%)	
Unknown^a^	2	0	2	
Number of metastases				0.095
1	1274 (75.3%)	197 (71.4%)	1077 (76.1%)	
> 1	417 (24.7%)	79 (28.6%)	338 (23.9%)	
*KRAS*				0.10
Wild type	710 (57.8%)	150 (62.5%)	560 (56.7%)	
Mutant	518 (42.2%)	90 (37.5%)	428 (43.3%)	
Unknown or not done^a^	463	36	427	
*NRAS*				> 0.9
Wild type	885 (95.2%)	176 (95.1%)	709 (95.2%)	
Mutant	45 (4.8%)	9 (4.9%)	36 (4.8%)	
Unknown or not done^a^	761	91	670	
*BRAF*				0.010
Wild type	931 (90.7%)	186 (86.1%)	745 (91.9%)	
Mutant	96 (9.3%)	30 (13.9%)	66 (8.1%)	
Unknown or not done^a^	664	60	604	
Mismatch repair				0.8
Deficient	42 (3.8%)	8 (3.6%)	34 (3.8%)	
Proficient	1068 (96.2%)	217 (96.4%)	851 (96.2%)	
Unknown or not done^a^	581	51	530	

Abbreviations: *BRAF*, B‐Raf proto‐oncogene, serine/threonine kinase; ECOG, Eastern Cooperative Oncology Group; IQR, interquartile range; *KRAS*, Kirsten rat sarcoma viral oncogene homologue; *NRAS*, neuroblastoma rat sarcoma viral oncogene homologue.

^a^ Unknown and missing variables have not been included in the statistical analysis.

### 3.2 Intention to Treat Versus Actual Treatments Offered

Table 2 and Table S2 outline the initial treatment intentions and the treatments ultimately received. The findings in Table 2 demonstrate an increased desire to offer active treatments to EOCRC patients than LOCRC patients at presentation (98.6% [219/222] vs. 92.1% [847/920]; *p* < 0.001). EOCRC patients were also significantly more likely to receive active treatments compared with LOCRC patients (96.0% [265/276] vs. 88.5% [1252/1415]; *p* < 0.001).

**TABLE 2:** Initial treatment plans and actual treatment sequences.

Treatment	Overall (*N* = 1691)	Age group		*p*
		≤ 50 years (*n* = 276; 16.3%)	> 50 years (*n* = 1415; 83.7%)	
Initial treatment plan				< 0.001
Palliation/best supportive care	76 (6.7%)	3 (1.4%)	73 (7.9%)	
Chemotherapy/chemoradiation	606 (53.0%)	135 (60.8%)	471 (51.2%)	
Surgery	460 (40.3%)	84 (37.8%)	376 (40.9%)	
Unknown	549	54	495	
Actual treatment sequence^a^				< 0.001
Chemotherapy only	512 (30.3%)	76 (27.5%)	436 (30.8%)	
Chemotherapy/biotherapy → surgery	277 (16.4%)	77 (27.9%)	200 (14.1%)	
Surgery only	158 (9.3%)	12 (4.4%)	146 (10.3%)	
Surgery → chemotherapy/biotherapy	570 (33.7%)	100 (36.2%)	470 (33.2%)	
Palliation/best supportive care/no treatment	174 (10.3%)	11 (4.0%)	163 (11.5%)	

^a^ Proportions in the > 50 years column do not add up to 100% due to rounding.

### 3.3 Overall Survival Differences Between the Two Cohorts and Effects of Treatment Sequence

Table S2 summarises the survival differences between the two cohorts as well as the impact of timing of the development of liver metastases (i.e., synchronous vs. metachronous), treatment sequence used and location of the primary cancer (i.e., right colon vs. left colon vs. rectum). Patients with EOCRC and liver‐only metastases had a longer median survival compared with patients with LOCRC (3.20 vs. 2.38 years; *p* < 0.001). There was also a significant difference in median survival among EOCRC patients with metachronous metastases compared with the LOCRC cohort (8.86 vs. 3.83; *p* < 0.001). While female sex was associated with worse survival in the LOCRC cohort, no impact was noted in the EOCRC cohort (Table S3).

In the EOCRC cohort, OS was affected by *KRAS* (HR, 2.38 [95% CI, 1.39–4.06]; *p* < 0.002), *NRAS* (HR, 3.57 [95% CI, 1.11–11.5]; *p* < 0.033) and *BRAF* V600E mutation status (HR, 2.83 [95% CI, 1.33–6.02]; *p* < 0.007), but not proficient MMR status. In the LOCRC cohort, OS was impacted by mutation of *KRAS* (HR, 1.55 [95% CI, 1.22–1.98]; *p* < 0.001) and *BRAF* (HR, 2.61 [95% CI, 1.79–3.81]; *p* < 0.001), but not mutation of *NRAS* or proficient MMR status (Table S3).

**FIGURE 1:**
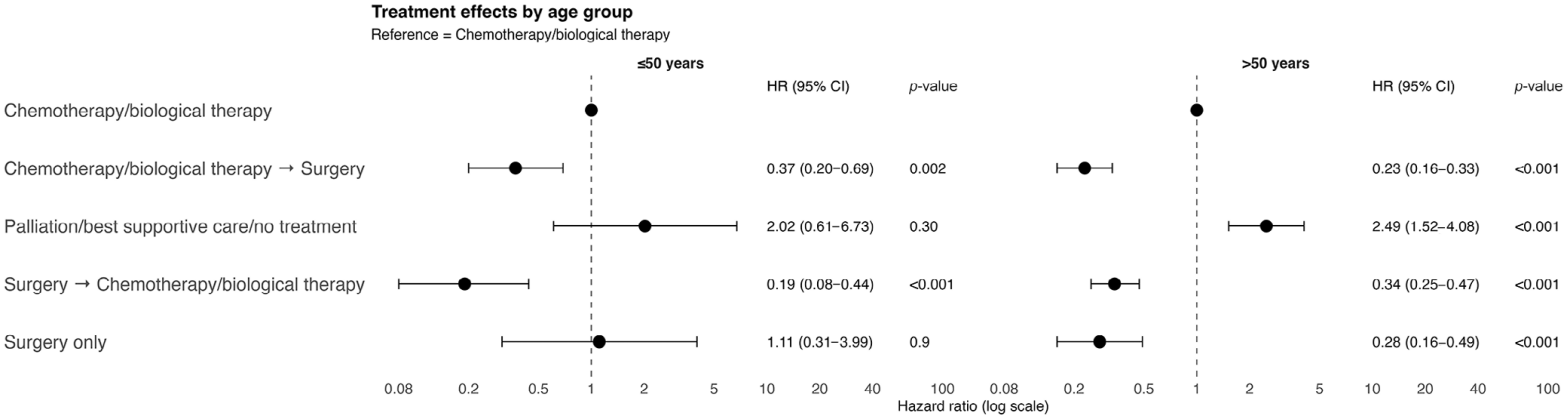
Forest plot of hazard ratios by treatment sequence for patients aged ≤ 50 years and > 50 years. CI, confidence interval; HR, hazard ratio.

**FIGURE 2:**
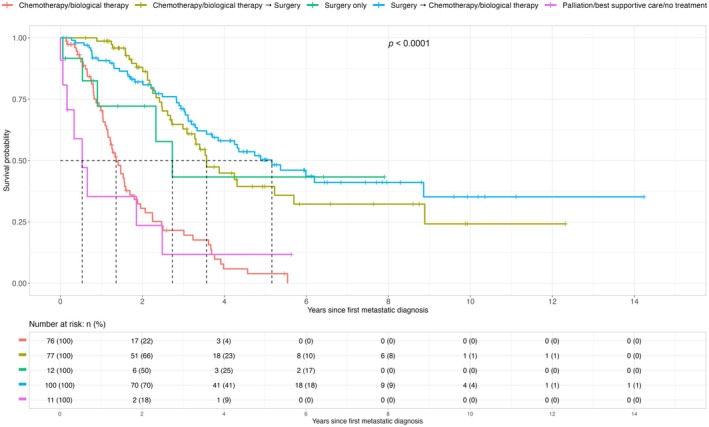
Overall survival by treatment group in patients aged ≤ 50 years.

**FIGURE 3:**
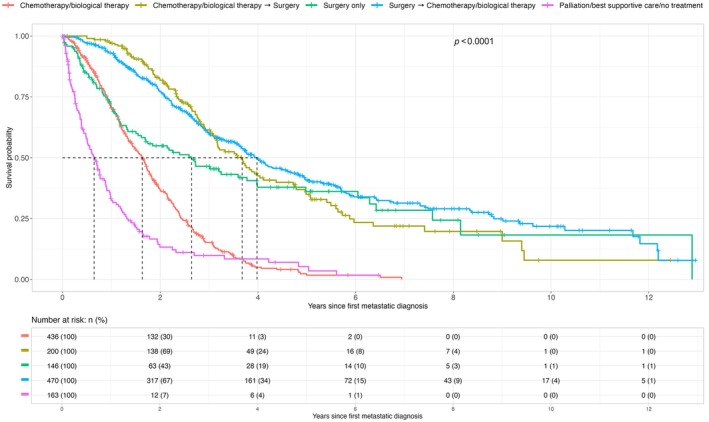
Overall survival by treatment group in patients aged > 50 years.

In both cohorts, a significant survival advantage was associated with patients having surgery first followed by adjuvant chemotherapy and/or biological therapies: HR, 0.19 (95% CI, 0.08–0.44; *p* < 0.001) for EOCRC and HR, 0.34 (95% CI, 0.25–0.47; *p* < 0.001) for LOCRC. This was more evident for those with synchronous metastases (median survival, 6.00 vs. 2.91 years) and those with left colon primary cancer (5.98 vs. 4.67 years) (Figures 1, 2, 3; Tables S2 and S3). In patients with metachronous metastases and in patients with right‐sided primaries, there appeared to be a different outcome between the EOCRC and LOCRC cohorts depending on the treatment sequence followed. The IPTW analysis was broadly consistent with the standard Cox model in patients aged > 50 years, but in patients aged ≤ 50 years, the survival advantages observed for surgery‐containing treatment sequences were attenuated and no longer statistically significant, while palliation/best supportive care was more strongly associated with poorer survival (Table 3).

**TABLE 3:** IPTW‐weighted Cox proportional hazards model: Hazard ratios and 95% CIs for treatment groups by age group (*n* = 1691).

Treatment group	Patients aged ≤ 50 years (*n* = 276)		Patients aged > 50 years (*n* = 1415)	
	HR (95% CI)	*p*	HR (95% CI)	*p*
Chemotherapy/biological therapy only	Reference	—	Reference	—
Chemotherapy/biological therapy → surgery	0.79 (0.25, 2.47)	0.70	0.32 (0.20, 0.51)	< 0.001
Surgery only	1.66 (0.41, 6.75)	0.50	0.58 (0.30, 1.10)	0.10
Surgery → chemotherapy/biological therapy	0.74 (0.26, 2.14)	0.60	0.41 (0.25, 0.67)	< 0.001
Palliation/best supportive care/no treatment	5.46 (1.44, 20.8)	0.013	2.78 (1.45, 5.34)	0.002

Abbreviations: CI, confidence interval; HR, hazard ratio; IPTW, inverse probability of treatment weighted.

## 4 Discussion

The findings of this study confirm the differences in sex distribution, mutation profile and primary site between the EOCRC and LOCRC cohorts reported by others previously. As expected, the younger patients tended to be more fit. Differences in *BRAF* mutation status have been reported in some other studies and in an earlier analysis of data from TRACC, which reported that this was limited to patients < 40 years old [18].

This study reflects the clinical practice of planning to treat younger patients with multimodality approaches from the outset. Novel findings from this population‐level dataset exploring survival among patients with CRC liver‐only metastases include the demonstration of variable outcomes between EOCRC and LOCRC with liver‐only metastases that are dependent on the treatment sequence, the timing of development of liver metastases (synchronous vs. metachronous) and the location of the primary CRC (right colon vs. left colon and rectum). These data also demonstrate an OS benefit for post‐operative chemotherapy in the resected Stage IV—a finding that has not yet been definitively shown in clinical trials.

Metastatic EOCRC is less likely to demonstrate the typical male predilection seen in CRC cohorts. This concurs with findings from the Surveillance, Epidemiology and End Results (SEER) database from the United States [19]. However, no impact of sex on OS was evident in this study. This is in contradiction to a recent finding using the United States SEER data that found a significantly improved survival among younger females with no difference in older females [19].

EOCRC liver‐only metastatic patients do not have an increased risk of associated comorbidities as evidenced by low Charlson comorbidity index scores. This finding is important considering the better outcomes achieved by upfront surgery, when feasible. Biological differences were seen in terms of the frequency of *BRAF* mutations (more common in the EOCRC cohort). Consistent with previous reports, both *RAS* and *BRAF* V600E mutations were associated with inferior OS across cohorts, [20, 21] reinforcing the importance of molecular stratification to optimise treatment selection and consideration of preoperative systemic therapy. However, data from the BREAKWATER trial suggest that incorporating targeted therapy (encorafenib and cetuximab) with chemotherapy may improve outcomes in *BRAF* V600E‐mutant disease, including EOCRC, with potential implications for management of this historically poor prognosis subgroup [22, 23].

In these data, we found that surgery (if feasible and with potential to be carried out with minimal morbidity) followed by adjuvant chemotherapy and/or biological therapies provides a significant survival advantage to all liver‐only metastatic CRC patients regardless of their age should they present with synchronous metastases and/or left colon primary cancer. In the IPTW‐weighted Cox analysis, treatment effect estimates in the > 50‐year group were broadly consistent with the conventional Cox model, suggesting that the original findings in this age stratum were relatively robust to adjustment for non‐random treatment allocation. In contrast, the ≤ 50‐year group showed greater changes after weighting. This is likely due to the much smaller sample size in the younger subgroup (*n* = 276), together with greater treatment selection imbalance and increased variability introduced by weighting. As a result, estimates in the younger group were less precise, as reflected by wider CIs, and should be interpreted cautiously.

The treatment of liver‐only metastatic CRC is nuanced with varying treatment algorithms tailored to the patient's disease and clinical characteristics [13, 24]. Owing to lack of data, current guidelines are unable to provide guidance about age‐based therapeutic surgical and systemic treatment protocols for EOCRC [25]. The findings of this study suggest that EOCRC patients with liver‐only metastases have a longer median survival compared with the LOCRC cohort (3.23 vs. 2.38 years; *p* < 0.001) with a significantly longer median survival for EOCRC patients with metachronous metastases (8.29 vs. 3.64 years; *p* < 0.001). These findings warrant consideration of a selective aggressive approach to managing CRC patients with liver‐only metastases based on disease burden, presence of comorbidities and an understanding of the patient's unique tumour biology.

## 5 Limitations

The strengths of our study are the prospective data collection, the large sample size and our focused analysis of patients with liver‐only disease. There are some limitations to our study.

Being an observational analysis, despite efforts to capture consecutive cases and regular auditing of data collection, the observational registry design remains susceptible to a degree of selection bias, residual confounding and data incompleteness inherent to real‐world datasets. This analysis is limited by the small number of eligible patients, which reduces statistical power and precision. Consequently, the study may have been underpowered to detect modest, but clinically meaningful, associations. Interpretation of survival differences between treatment sequence groups should be undertaken with caution. Patients must survive long enough to receive subsequent treatments, such as surgery or chemotherapy, which may introduce immortal time bias. Furthermore, surgical resection is preferentially offered to patients with more favourable disease characteristics or technically resectable metastases, which may introduce treatment selection bias. As a result, the observed survival differences between treatment groups likely reflect a combination of treatment patterns and underlying patient or disease characteristics rather than causal treatment effects.

Molecular profiling data were unavailable for the entire cohort—even for those patients who were included from 2015 onwards (a point in time when first‐line cetuximab was first available in Australia and testing for *RAS* was used to guide initial treatment decisions). Missing data, particularly in molecular variables, may have introduced bias if the missingness was not random. As analyses were based on available data, the potential impact of incomplete covariate information on the observed associations should be considered when interpreting the results. Variability in *RAS* and *BRAF* mutation and MMR proficiency testing between the two cohorts is also evident. The more aggressive approach (diagnostic and therapeutic) to the EOCRC cohort is evident by a higher uptake of molecular testing in the EOCRC group. The more recent increase in *RAS* testing mirrors global trends in reflex testing [26], although evidence exists to support low rates of testing for *RAS*, *BRAF* and microsatellite instability and MMR status in the United States [27]. Treatment practices and molecular testing evolved during the study period, which may have influenced treatment selection and outcomes.

## 6 Conclusion

The rising burden of EOCRC globally demands a better understanding of its biology and clinical behaviour [28]. Differences in sex distribution, left sidedness, *BRAF* mutation expression and mutational impact on OS suggest biological variations between EOCRC and LOCRC that need to be considered when planning treatment strategies, including resectional surgery and liver transplantation [24]. The treatment approach, particularly earlier timing of surgery, may provide improved survival outcomes, when feasible.

## Author Contributions

**Savio G. Barreto:** conceptualisation, project administration of the study. **Savio G. Barreto, Christos S. Karapetis** and **Peter Gibbs:** methodology. **Angus Campbell:** data curation. **Shahid Ullah:** software and investigation. **Savio G. Barreto and Shahid Ullah:** formal analysis. **Savio G. Barreto and Shahid Ullah:** writing – original draft. All authors: writing – review and editing.

## Funding

Savio G. Barreto was funded by a Flinders Foundation Grant (49358025), a National Health and Medical Research Council Ideas Grant (2021009), Pankind 21.R7.INV.CB.UOSA.6.2, the CUREator scheme via Brandon BioCatalyst, the Hospital Research Fund and a Southern Adelaide Local Health Network Enquiry Grant Round, and royalties received from Springer Nature and CRC press for editing books in surgery not related to this study.

## Conflicts of Interest

The authors declare no conflicts of interest.

## Supporting information


**Table S1:** STROBE Statement for cohort studies.
**Table S2:** Median survival times in years (95% CI) between two age groups (*n* = 1691).
**Table S3:** Hazard ratios (HR) and 95% CI (Cox Proportional hazard model) for treatment group between two age groups (*n* = 1691).

## Data Availability

Data, code and materials are available upon request.
