# Supplementary material for: Early‐Onset Colorectal Cancer With Liver‐Only Metastases: A Retrospective Cohort Study Integrating Prospectively Collected Real‐World Clinical and Molecular Data From an Australian National Database (2009–2024) to Guide Treatment Planning
Source: Med J Aust. 2026 Aug 18;224(8):e70266. doi: 10.5694/mja2.70266 (PMC13487442; doi:10.5694/mja2.70266)
Supplement: Supplementary file 1 [file MJA2-224-0-s001.pdf]

Supporting Information

Supplementary material

This appendix was part of the submitted manuscript and has been peer reviewed.
It is posted as supplied by the authors.

Appendix to: S. G. Barreto, C. S. Karapetis, S. Ullah, et al. Early-Onset Colorectal Cancer With Liver-Only Metastases: A Retrospective Cohort Study Integrating Prospectively Collected Real-World Clinical and Molecular Data From an Australian National Database (2009–2024) to Guide Treatment Planning. *Med J Aust* 2026; doi: 10.5694/mja2.70266.

Table S1: STROBE Statement for cohort studies

	Item No	Recommendation	Page No
**Title and abstract**	1	(*a*) Indicate the study's design with a commonly used term in the title or the abstract	1
		(*b*) Provide in the abstract an informative and balanced summary of what was done and what was found	2-3
**Introduction**			
Background/rationale	2	Explain the scientific background and rationale for the investigation being reported	5
Objectives	3	State specific objectives, including any prespecified hypotheses	6
**Methods**			
Study design	4	Present key elements of study design early in the paper	6
Setting	5	Describe the setting, locations, and relevant dates, including periods of recruitment, exposure, follow-up, and data collection	6
Participants	6	(*a*) Give the eligibility criteria, and the sources and methods of selection of participants. Describe methods of follow-up<br>(*b*) For matched studies, give matching criteria and number of exposed and unexposed	6-7
Variables	7	Clearly define all outcomes, exposures, predictors, potential confounders, and effect modifiers. Give diagnostic criteria, if applicable	7-8
Data sources/ measurement	8*	For each variable of interest, give sources of data and details of methods of assessment (measurement). Describe comparability of assessment methods if there is more than one group	8-9
Bias	9	Describe any efforts to address potential sources of bias	9
Study size	10	Explain how the study size was arrived at	n.a.
Quantitative variables	11	Explain how quantitative variables were handled in the analyses. If applicable, describe which groupings were chosen and why	n.a.
Statistical methods	12	(*a*) Describe all statistical methods, including those used to control for confounding<br>(*b*) Describe any methods used to examine subgroups and interactions<br>(*c*) Explain how missing data were addressed<br>(*d*) If applicable, explain how loss to follow-up was addressed<br>(*e*) Describe any sensitivity analyses	8-9
**Results**			
Participants	13*	(a) Report numbers of individuals at each stage of study—eg numbers potentially eligible, examined for eligibility, confirmed eligible, included in the study, completing follow-up, and analysed<br>(b) Give reasons for non-participation at each stage<br>(c) Consider use of a flow diagram	10
Descriptive data	14*	(a) Give characteristics of study participants (eg demographic, clinical, social) and information on exposures and potential confounders<br>(b) Indicate number of participants with missing data for each variable of interest<br>(c) Summarise follow-up time (eg, average and total amount)	10 Table 1
Outcome data	15*	Report numbers of outcome events or summary measures over time	10-11 Suppl Tables 1-2 Table1-2, 3 Figures 1-3

Main results	16	(*a*) Give unadjusted estimates and, if applicable, confounder-adjusted estimates and their precision (eg, 95% confidence interval). Make clear which confounders were adjusted for and why they were included<br>(*b*) Report category boundaries when continuous variables were categorized<br>(*c*) If relevant, consider translating estimates of relative risk into absolute risk for a meaningful time period	10-11 Suppl Tables 1-2 Table1-2, 3
Other analyses	17	Report other analyses done—eg analyses of subgroups and interactions, and sensitivity analyses	n.a.
**Discussion**			
Key results	18	Summarise key results with reference to study objectives	12
Limitations	19	Discuss limitations of the study, taking into account sources of potential bias or imprecision. Discuss both direction and magnitude of any potential bias	15
Interpretation	20	Give a cautious overall interpretation of results considering objectives, limitations, multiplicity of analyses, results from similar studies, and other relevant evidence	12-14
Generalisability	21	Discuss the generalisability (external validity) of the study results	12-14
**Other information**			
Funding	22	Give the source of funding and the role of the funders for the present study and, if applicable, for the original study on which the present article is based	Title page

*Give information separately for exposed and unexposed groups.

Note: An Explanation and Elaboration article discusses each checklist item and gives methodological background and published examples of transparent reporting. The STROBE checklist is best used in conjunction with this article (freely available on the Web sites of PLoS Medicine at http://www.plosmedicine.org/, Annals of Internal Medicine at http://www.annals.org/, and Epidemiology at http://www.epidem.com/). Information on the STROBE Initiative is available at http://www.strobe-statement.org.

Table S2. Median survival times in years (95% CI) between two age groups (n=1,691)

Cancer sites	Patient groups			
	(<=50 years)		(>50 years)	
	N	Median Survivals (95% CI)	N	Median Survivals (95% CI)
Overall	276	3.20 (2.72-3.69)	1,415	2.38 (2.23-2.57)
Treatment group				
Chemotherapy/biological therapy only	76	1.36 (1.16-1.80)	436	1.63 (1.44 – 1.74)
Chemotherapy/biological therapy → surgery	77	3.57 (3.08-8.89)	200	3.68 (3.15 – 4.11)
Surgery only	12	2.73 (0.90-NA)	146	2.64 (1.69 – 3.98)
Surgery → chemotherapy/biological therapy	100	5.16 (3.70-NA)	470	3.98 (3.63 – 4.61)
Palliation/best supportive care/no treatment	11	0.53 (0.16-NA)	163	0.65 (0.50 – 0.86)
Synchronous				
Chemotherapy/biological therapy only	65	1.36 (1.16-1.88)	288	1.42 (1.25 – 1.65)
Chemotherapy/biological therapy → surgery	58	3.57 (3.28-NA)	124	2.83 (2.65 – 3.58)
Surgery only	1	NA	20	1.18 (0.88 – 1.85)
Surgery → Chemotherapy/biological therapy	19	6.00 (4.25-NA)	72	2.91 (2.25 – 4.30)
Palliation/best supportive care/no treatment	4	0.33 (0-NA)	91	0.39 (0.30 – 0.59)
Metachronous				
Chemotherapy/biological therapy only	11	1.34 (0.81 – NA)	148	2.00 (1.73 – 2.28)
Chemotherapy/biological therapy → surgery	19	2.72 (2.48 – NA)	75	4.99 (4.37 – NA)
Surgery only	11	2.73 (0.90 – NA)	126	3.25 (2.26 – 6.42)
Surgery → Chemotherapy/biological therapy	81	4.74 (3.35 – NA)	397	4.05 (3.72 – 4.93)
Palliation/best supportive care/no treatment	7	1.85 (0.16 – NA)	72	1.23 (0.76 – 1.65)
Rectal				
Chemotherapy/biological therapy only	25	1.34 (1.13 – 2.47)	134	1.71 (1.35 – 1.95)
Chemotherapy/biological therapy → surgery	37	NA (3.56 – NA)	92	3.17 (2.80 – 4.11)
Surgery only	1	NA	26	3.78 (2.72 – NA)
Surgery → Chemotherapy/biological therapy	15	3.70 (2.94 – NA)	65	5.69 (4.12 – NA)
Palliation/best supportive care/no treatment	4	1.85 (1.85 – NA)	53	0.88 (0.70 – 1.47)
Left colon				
Chemotherapy/biological therapy only	33	1.70 (1.16 – 3.61)	155	1.67 (1.45 – 1.96)
Chemotherapy/biological therapy → surgery	26	3.09 (2.42 – NA)	74	3.73 (3.18 – 5.97)
Surgery only	6	2.33 (2.32 – NA)	61	2.71 (1.20 – NA)
Surgery → Chemotherapy/biological therapy	59	5.98 (4.35 – NA)	215	4.67 (3.93 – 5.70)
Palliation/best supportive care/no treatment	4	0.41 (0.06 – NA)	54	0.50 (0.39 – 0.86)
Right colon				
Chemotherapy/biological therapy only	18	1.26 (0.80 – NA)	126	1.30 (1.16 – 1.71)
Chemotherapy/biological therapy → surgery	14	2.68 (1.74 – NA)	32	4.04 (2.72 – NA)
Surgery only	5	NA (0.90 – NA)	57	1.55 (1.02 – 3.25)
Surgery → Chemotherapy/biological therapy	26	3.20 (2.83 – NA)	181	2.75 (2.42 – 3.01)
Palliation/best supportive care/no treatment	3	0.33 (0- NA)	50	0.48 (0.27 -1.15)
Duration between primary and metastasis diagnosis				
<6 months	15	2.94 (2.12-NA)	73	2.23 (1.80 – 3.69)
>= 6 months	38	NA (2.48-NA)	402	3.64 (3.15 – 4.67)

Abbreviations: CI – confidence interval; NA – not available

Table S3. Hazard ratios (HR) and 95% CI (Cox Proportional hazard model) for treatment group between two age groups (n=1,691).

	(<=50 years)		(>50 years)	
	N=276		N=1,415	
	HR (95% CI)	P-value	HR (95% CI)	P-value
**Treatment group**				
Chemotherapy/biological therapy only	Reference	-	Reference	-
Chemotherapy/biological therapy → surgery	0.37 (0.20, 0.69)	0.002	0.23 (0.16, 0.33)	<0.001
Surgery only	1.11 (0.31, 3.99)	>0.9	0.28 (0.16, 0.49)	<0.001
Surgery → chemotherapy/biological therapy	0.19 (0.08, 0.44)	<0.001	0.34 (0.25, 0.47)	<0.001
Palliation/best supportive care/no treatment	2.02 (0.61, 6.73)	0.3	2.49 (1.52, 4.08)	<0.001
**Sex (**Female vs Male (ref))	0.93 (0.56, 1.54)	0.8	1.33 (1.05, 1.68)	0.016
**Charlson's index**				
0	Reference	-	Reference	-
1	2.22 (1.06, 4.66)	0.034	1.43 (1.09, 1.87)	0.009
2+	0.45 (0.09, 2.26)	0.3	0.97 (0.70, 1.34)	0.8
**Primary sites**				
Left colon	Reference	-	Reference	-
Right colon	1.09 (0.60, 1.99)	0.8	1.11 (0.85, 1.47)	0.4
Rectum	0.65 (0.35, 1.23)	0.2	1.05 (0.78, 1.41)	0.7
**Stage at diagnosis** (Metachronous (ref) vs Synchronous)	0.69 (0.30, 1.59)	0.40	1.88 (1.34, 2.63)	<0.001
**Metastases** (1 (ref) vs >1)	2.38 (1.41, 4.04)	<0.001	1.13 (0.88, 1.45)	0.3
**KRAS** (Mutant vs Wild Type (ref))	2.38 (1.39, 4.06)	0.002	1.55 (1.22, 1.98)	<0.001
**NRAS** (Mutant vs Wild Type (ref))	3.57 (1.11, 11.5)	0.033	1.17 (0.64, 2.13)	0.6
**BRAF** (Mutant vs Wild Type (ref))	2.83 (1.33, 6.02)	0.007	2.61 (1.79, 3.81)	<0.001
**MMR** (Proficient vs deficient (ref))	7.29 (0.78, 68.1)	0.082	1.29 (0.62, 2.65)	0.5

Abbreviations: BRAF - ***B-Raf proto-oncogene, serine/threonine kinase; CI – confidence interval;*** *ECOG – Eastern Cooperative Oncology Group; IQR – interquartile range; KRAS -* ***Kirsten Rat Sarcoma viral oncogene homolog;*** *NRAS -* ***Neuroblastoma RAS viral oncogene homolog;*** *MMR –* ***Mismatch repair***
